# Process evaluation of a Germany-wide complex intervention to prevent dysregulated screen time in under threes: a RE-AIM-approach

**DOI:** 10.3389/fped.2025.1382428

**Published:** 2025-05-02

**Authors:** Juliane Schemmer, David Martin, Hanno Krafft, Tobias Maurer, Anke Emgenbroich, Sean Monks, Silke Schwarz

**Affiliations:** ^1^ Institute for Integrative Medicine, Chair of Medical Theory, Integrative and Anthroposophic Medicine, Department of Human Medicine, Faculty of Health, University of Witten/Herdecke, Herdecke, Germany; ^2^ Interprofessional Graduate School Integrative Medicine and Health, Health Department, University of Witten/Herdecke, Witten, Germany; ^3^ University Clinic for Pediatric and Adolescent Medicine, University of Tübingen, Tübingen, Germany; ^4^ BVKJ-Service GmbH, Cologne, Germany; ^5^ Monks – Ärzte im Netz GmbH, Munich, Germany

**Keywords:** birth cohort study, screen time, pediatrics, outpatient, complex intervention, randomized controlled trial, RE-AIM, healthcare research

## Abstract

**Background:**

Children's current screen time is well above current recommendations and is associated with many health consequences in the first years of life.

**Methods:**

The complex intervention study “Screen free till 3” introduced parent education to a regular examination of 6-month-old children in outpatient pediatric practices. Pediatric practices were cluster-randomized in a 2:1 ratio (intervention group:control group). 2,581 pediatric practices received the intervention materials by cold call and participated by self-selection. The study includes a process evaluation that examines the implementation process. In this article, four different quantitative methods of the process evaluation are evaluated according to the RE-AIM scheme.

**Result:**

33.4% of pediatric practices confirmed their participation in the study. 10,391 parents took part in the pre-interventional app-based parent survey. 151 interested institutions contacted the research team by email. The majority (84.1%) asked to take part in the study. 518 pediatric practices took part in a telephone survey, of which 87.2% said that they are supported by the intervention materials and 91.6% would recommend the project to others.

**Discussion:**

The RE-AIM analysis shows a high reach of parents via the app. The high adoption by pediatric practices and other institutions characterizes the relevance of the topic as well as the innovation of the study materials. After one and a half years, the intervention is firmly integrated into the structures of pediatric practices in Germany.

**Trial register number:**

https://drks.de/search/en/trial/DRKS00032258, DRKS00032258.

## 1 Introduction

Screen exposure in early childhood can be associated with developmental impairments and health problems. The average screen time of children under 5 years of age worldwide ranges from 0.1 to 5 h in 2019 (1). A survey conducted among German mothers in 2022 revealed that the average screen time of 3- to 5-year-olds was 93 min for boys (77 min of television) and 83 min for girls (71 min of television) (2).

Specific risks associated with screen media exposure in early childhood are: delayed language development (3–5), learning problems, social-emotional delays, hyperactivity, inattention, aggressive and antisocial behavior (4). Early screen time is also negatively associated with fine motor and gross motor development (4). Positive correlations have been found between increased screen time and obesity (6, 7), insulin resistance and type 2 diabetes mellitus (8, 9), sleep problems (4) and myopia in preschool children (6, 10). Structural differences in the brain (11, 12) and altered cortical electroencephalography (EEG) activity affecting executive functions have been associated with increased screen use in preschool children (13).

There are guidelines to reduce the screen time of infants and toddlers. The World Health Organization, recommend no screens for children <2 years and a maximum of 60 min per day for children aged 2–4 years (14). The German AWMF guideline recommends screen time restriction for the first three years of life and a maximum daily screen time of 30 min per day for 3- to 6-year-olds (15). However, an observational study in France from 2022 shows that only a small proportion (13.5%) of parents adhere to the no screen guideline. Socio-demographic factors and high levels of screen time among parents were associated with non-compliance with the guideline (16). Qualitative research shows that some parents do not know the guidelines (17), are uninformed about the dangers of screen time in early childhood and instead see digital devices as an inevitable and necessary part of life (18). Parents use screens to keep children occupied, to keep them calm, and to help with difficulties such as mealtimes or teething (19). However, they also report that screen time of children and parents leads to conflicts between parents and children, to tantrum and aggression in children, and to conflicts between parents (20, 21).

In Germany, there is currently no intervention to prevent excessive screen media use by children under 3 years of age. Experts are therefore calling for more prevention and support services (22). Parent education may help to prevent or reduce children's screen time (23–25). However, previous interventions to reduce screen time in the first years of life have shown inconsistent effects on children's screen time (26). A process evaluation should evaluate the implementation process of such an intervention in terms of content and context (27). This increases effectiveness and provides an example for the development of further complex interventions.

## 2 Materials and methods

### 2.1 The complex intervention study

To our knowledge, the “Screen free till 3” (BB3)-study is the world's largest intervention study to date on the prevention of screen media use in children under the age of three (28). The University of Witten/Herdecke initiated the study in cooperation with the Professional Association of Pediatricians in Germany (BVKJ) and BVKJ-Service GmbH (BVKJ-S) in 2022.

The cluster-randomized controlled trail evaluates the complex intervention with a mixed-methods design. Pediatric practices were randomized 2:1 (intervention group:control group). In 2022, the intervention group (*n* = 2,581 pediatric practices) received the intervention materials in cold calling. Participation was by self-selection. The control group did treatment as usual. Parents of children from intervention and control practices take part in the longitudinal parent survey. The parent survey in the BVKJ's practice app examines screen time, time in nature and children's development from six months to three years of age. In addition, a mixed-methods process evaluation evaluated the implementation process of the complex intervention and the contextual factors.

### 2.2 Study design of the process evaluation

The process evaluation was based on the logical model of the BB3 intervention and the MRC Guidance (29) for the process evaluation of complex interventions. The basic aim of the process evaluation was to examine the implementation process of the intervention study. The objectives were as follows: (1) To evaluate the implementation process in pediatric practices. (2) To evaluate the reach and effectiveness of the intervention in relation to parents. The RE-AIM scheme (Reach, Effectiveness, Adoption, Implementation, Maintenance) (30, 31) represented the implementation process of the intervention (Table 1).

**Table 1:** Framework of the process evaluation of BB3 intervention study.

RE-AIM	Project definition	Data type and source
Implementation process		
Reach	Absolute number, proportion and representativeness of participants in the app survey; use of the project website	App survey, website analysis
Effectiveness	Economic effects of the intervention on the pediatric practices and other reasons for or against participation; behavioral prevention or change of the parents in group comparison; development parameters of the children in group comparison	App survey
Adoption	Absolute number, proportion, and representativeness of participating practices and of interested institutions	Practice documentation, website analysis
Implementation	Fidelity, protocol-compliant implementation in practices; applicability of the intervention; adaptations made to interventions and implementation strategies	Telephone survey, practice documentation
Maintenance	Extent to which the intervention becomes part of the routine organizational practices and guidelines and is maintained at the individual level	Practice documentation app survey

### 2.3 Data collection and analysis

#### 2.3.1 Data collection

##### 2.3.1.1 Practice documentation

The practice documentation documents the participating and non-participating intervention practices and the communication process.

##### 2.3.1.2 Telephone survey

In 2022, a call centre conducted a telephone survey with a random sample of 800 pediatric practices from the intervention group. This comprised eight questions about the materials and the implementation of the intervention.

##### 2.3.1.3 Parent survey

The longitudinal parent survey in the practice app includes children born in 2022. Parents fill out a questionnaire at four points in time according to the age of the children (6 months, 1 year, 2 years and 3 years). The first questionnaire is pre-interventional. The following questionnaires are post-interventional. The survey records the daily screen time and the child's development.

##### 2.3.1.4 Website analysis

The website analysis evaluated the frequency of use of the project website, registrations for the parent newsletter and inquiries via the contact form on the project website.

#### 2.3.2 Data analysis

The data from the quantitative surveys were exported to Excel and Statistical Package for Social Sciences (SPSS) for analysis. The distributions of the collected data were described separately for the different collectives using suitable descriptive statistical parameters.

## 3 Results

### 3.1 Reach

The first pre-interventional parent survey (June 2022 to October 2023) gave 36,431 parents the opportunity to participate. There was the questionnaire on sociodemographic (quest 1) and the questionnaire with questions about screen time and child development (quest 2). 28.5% (10,391) completed quest 1 and 17.8% (6,469) completed quest 2. Participants were mothers (94.1%), fathers (5.7%) and foster parents or others (0.2%). This data came from 786 practices throughout Germany. We have published the more detailed results of the baseline survey separately (28).

The project website received 45,883 visits from June 2022 to December 2023. 94% of the visits came from Germany, followed by Austria, Switzerland, Belgium, and the USA. On average, visitors performed 5.4 actions per page. Over the entire period, the homepage had 38.5% of total views. The most clicked content after the homepage was background information on the study (23.5%), tips and tricks (7.5%) and frequently asked questions (7.4%). The English-language homepage had 2.4% of total views. The information pages for pediatric practices had 3.7% of visits. Until December 2023, 699 readers have subscribed to the email newsletter.

### 3.2 Effectiveness

We are evaluating the effectiveness of the intervention via the parent survey as well as parent and expert interviews. We will publish the results separately.

### 3.3 Adoption

In May 2022, the 2,581 pediatric practices in the intervention group received the first package with intervention materials and study information in cold calls. Self-selection without exclusion criteria by the pediatric practices determined the actual sample size. At the time of writing this manuscript, 33.4% of pediatric practices (861) had already confirmed their participation in the study. The relative proportion of participating practices in relation to non-participating intervention practices per federal state averages 33.3% (median = 33.0%) describes a symmetrical distribution. Nine practices from the intervention group (0.3%) informed the research team by email that they did not wish to participate in the study (Table 2).

**Table 2:** Germany-wide distribution of pediatric practices from the intervention group participating in self-selection by state.

Distribution of pediatric practices from the intervention group				
	Randomized intervention group	Participation confirmed	% of participation confirmed	% by intervention group by state
Total	**2,581**	**861**	**100** **.** **0**	**33** **.** **4**
Baden-Wuerttemberg	351	134	15.6	38.2
Bavaria	448	157	18.2	35.0
Berlin	153	44	5.1	28.8
Brandenburg	93	12	1.4	12.9
Bremen	21	6	0.7	28.6
Hamburg	64	23	2.7	35.9
Hesse	176	58	6.7	33.0
Mecklenburg-Vorpommern	30	14	1.6	46.7
Lower Saxony	199	86	10.0	43.2
North Rhine-Westphalia	568	169	19.6	29.8
Rheinland-Pfalz	107	31	3.6	29.0
Saarland	33	10	1.2	30.3
Saxony	125	42	4.9	33.6
Saxony-Anhalt	79	25	2.9	31.6
Schleswig-Holstein	80	26	3.0	32.5
Thuringia	54	24	2.8	44.4
	Randomized intervention group	Participation refused		% by intervention group by state
Total		**9**		**0** **.** **3**
Baden-Wuerttemberg	351	1		0.3
Bavaria	448	1		0.2
Hamburg	64	1		1.6
Lower Saxony	199	2		1.0
North Rhine-Westphalia	568	3		0.5
Saxony	125	1		0.8

Bold values: total sample; randomized intervention group: number of practices that were randomized to the intervention group; participation confirmed: number of practices that have confirmed their participation; participation refused: number of practices that have refused to participation; % by intervention group by state: proportion of practices from the intervention group, per state participating or not participating.

By December 11, 2023, 151 people interested in the project had sent an email request. These were doctors from the control group (51.7%), child day care and schools (10.6%), family support and counseling (9.9%), offices and administration (8.6%), therapy (7.3%) and clinics and others (6.0%). The reasons for the inquiries were requests for material and information on the study (84.1%), cooperation and support for the study, e.g., through public relations work (9.3%), suggestions for improvement such as translation into other languages (3.3%), requests for speakers (2.6%) and other (0.7%).

### 3.4 Implementation

In July 2022, a telemarketing agency contacted a random sample of 800 pediatric practices from the intervention group. Five hundred and eighteen pediatrician practices participated. The medical assistants answered most frequently (96.0%). 87.3% (452) of pediatric practices confirmed that they had received the BB3-intervention materials. 91.4% (413) of respondents confirmed that they had already advised against screen media in early childhood before the start of the BB3 intervention, 4.6% (21) did not. 87.2% (394) of pediatric practices reported that the intervention helped them with parent counseling in the practice. This was not the case for only 7.5% (34). 91.6% (414) of respondents confirmed that they would recommend the project to other pediatric practices.

### 3.5 Maintenance

During the study period, we sent intervention material and reminders to the intervention practices several times by post and e-mail. Pediatric practices also had the option of reordering intervention materials via website. By December 2023, 128 pediatric practices had reordered further study materials.

## 4 Discussion

With a participation rate of at least 33.4% of the pediatric practices invited to the study, the pediatricians accepted the BB3 intervention study very well. The expected recruitment rate of general practitioner practices for outpatient research projects is 3% to 4% (32). The high participation rate with a symmetrical, Germany-wide distribution is possibly due to the urgency of the topic “digitalization in early childhood”. High number of e-mail inquiries from the control group and other institutions underlines the relevance of the topic. “Screen free till 3” can also be implemented and expanded through family support and counseling, daycare and schools, therapy, offices, and clinics. A qualitative analysis of an intervention study with parent education on screen time found that parents trust the information provided by a health advisor (33). In addition, a study with parent education in kindergartens showed a reduction in screen time for children with pre-interventional screen time of ≥2 h per day (34). Parents seem to trust the information from professionals regarding screen use and limit the screen time for their children.

The practice app was used to reach 10,391 parent-child pairs for participation in the parent survey, which corresponds to around 1.4% of all children born in Germany in 2022 (738,819) (35). The study has a large birth cohort that evaluates screen use and child development longitudinally up to the age of three. Many studies only measure the time and devices children use (36–38). Increased screen time by parents may be a risk factor for non-compliance with screen time guidelines (16). In addition, parental use of digital devices hinders interaction between parent and child (20, 21). The BB3 study examines the effect of parents' screen time in presence of the child and internet behavior on child's screen time and development (28).

A multicenter cross-sectional study in France from 2021 showed that only 22.7% of parents received education about screen exposure in early childhood from their doctor. Of these, 53.1% did so on their own initiative (39). The intervention materials enables structured parent education at every regular examination. The intervention materials supports the pediatricians. The majority of the pediatric practices contacted said they would recommend the project to others. This underlines the acceptance and practicability by pediatricians. High screen time in infants and toddlers is associated with parental education and other sociodemographic factors (1, 16, 40). An intervention via the pediatrician for a regular examination reaches almost all parents in Germany and can potentially minimize this effect.

Qualitative studies have already found that parents are often unaware of the risks of screen media exposure, see the use of digital devices as integrated and relevant in everyday life, and also have difficulty reducing their child's screen time (17, 18, 20). The qualitative part of the process evaluation separately examines which of these factors also apply to German families. In addition, the qualitative analysis examines whether these factors can be reduced through early intervention at the age of 6 months. In interviews, parents and grandparents report that the use of digital media often leads to conflicts with small children or tantrum and aggression (20). The no screen guideline may reduce these effects.

There are possible limitations with regard to the results. The reported participation rate may differ from the real participation rate. It is possible that many pediatric practices use the intervention materials without confirmation of participation, as they regularly receive materials without ordering them. The majority of respondents to the telephone survey were medical assistants. Especially in large practices, it can happen that a person is reached on the phone who is not involved in the BB3-intervention study in their area of activity. Consequently, the results of the telephone survey (participation, recommendation, etc.) may differ from the real results.

In summary, the BB3 intervention has been successfully implemented in many pediatric practices in Germany based on an assessment according to the RE-AIM scheme. After one and a half years, the intervention had a wide reach among pediatricians, and was noticed and endorsed by numerous institutions. The large number of interested institutions reflects the relevance of such a project and offers many opportunities to firmly establish the BB3 intervention. The process evaluation also showed how the practice app achieved a high number of participating parents.

## Data Availability

The datasets presented in this article are not readily available because they are not anonymized (in the case of the practice documentation) and we received them from the call center (in the case of the telephone survey). Requests to access the datasets should be directed to juliane.schemmer@uni-wh.de.
